# Analysis of EGFR signaling pathway; miRNAs and inflammatory biomarkers in a high-risk oral cancer population in Pakistan - An exploratory study

**DOI:** 10.1371/journal.pone.0340264

**Published:** 2026-02-23

**Authors:** Namrah Anwar, Shahid Pervez, Tariq Moatter, Mitchell Stark, Qurratulain Chundriger, Tazeen Saeed Ali, Sohail Awan, Annika Antonsson

**Affiliations:** 1 Department of Pathology and Laboratory Medicine, Aga Khan University Hospital, Karachi, Pakistan; 2 Faculty of Science and Technology, University of Central Punjab, Lahore, Pakistan; 3 Frazer Institute, The University of Queensland, Dermatology Research Centre, Brisbane, Queensland, Australia; 4 Royal Blackburn Teaching Hospital East Lancashire Hospitals NHS Trust, Blackburn, UK; 5 School of Nursing and Midwifery, Aga Khan University Hospital, Karachi, Pakistan; 6 Department of Otolaryngology, Head and Neck Surgery, Aga Khan University Hospital, Karachi, Pakistan; 7 Department of Population Health, QIMR Berghofer Medical Research Institute, Brisbane, Queensland, Australia; University of Hong Kong, HONG KONG

## Abstract

Oral cancer has high morbidity rates in the Asian region, specifically Pakistan. However, little insight is available regarding the molecular pathogenesis and inflammatory biomarkers of this preventable cancer. This study determined the association of EGFR, NFκB, COX-2, and miRNAs expression with the chewing habits and high-risk human papillomavirus (HR-HPV) infection in oral cancer patients. Formalin-fixed paraffin-embedded blocks (FFPE) (n = 50) were analyzed for transcriptional expression of EGFR, NFκB, and COX-2 by qPCR and COX-2 protein expression was checked by immunohistochemistry (IHC). Array profiling of ~2500 miRNAs was performed on nine samples, and five miRNAs were selected for validation from this profiling data. Appropriate statistical tests were applied to check the association of EGFR., NFκB, COX-2, and miRNAs with chewing and HR-HPV status, (p < 0.05 and 95% CI). Of the 50 samples, transcriptional expression of EGFR was observed in 13 (26%), NFκB in 11 (22%), and COX-2 in 17 (34%) samples, and the majority were chewers. EGFR and COX-2 expression was significantly associated with chewing gutka (the most carcinogenic form of chewing substance). A total of 281 miRNAs were dysregulated in miRNA profiling, and in validation, miR-222-3p was significantly downregulated in chewers expressing EGFR, NFκB, and COX-2 compared to non-chewers (p < 0.05). HR-HPV positivity was not correlated with miRNAs. This study suggests a significant association of chewing habits with EGFR and COX-2 expression. In addition, the molecular pathogenesis of OSCC suggests the substantial interplay of NFκB, COX-2, EGFR and miRNAs in chronic chewers, irrespective of HR-HPV involvement.

## Introduction

Amongst all cancers, lip and oral cavity cancers have the highest prevalence and morbidity in South Asian countries (Bangladesh, Sri Lanka, India, and Pakistan) [1]. In Pakistan, according to the most recent figures, cancer of the lip and oral cavity stands second on the list when both genders combined, with 13% new cases [2]. As per the National Cancer Registry (NCR) of Pakistan, oral cancer (OC) is leading in males and third most common in females, with 11.6% and 4.9% cases, respectively [3]. The most common histological type of OC is Oral Squamous Cell Carcinoma (OSCC) and etiology includes chewing habits (betel quid (BQ), areca nut (AN), and gutka), as well as molecular pathogenesis.

The EGFR signaling pathway is one of the most studied molecular mechanisms in OSCC. EGFR is a receptor tyrosine kinase (RTKs) localized on the plasma membrane, which upon ligand binding, triggers autophosphorylation, and multiple intracellular signaling cascades are activated. It is one of the prominent targets for cancer therapy in Oral Cancers [4]. EGFR activates RAS-RAF-MAPK, PI3K/ AKT-mTOR-NFκB, or JAK-STAT pathways. This cascade activation results in various cellular activities, including modulation of apoptosis, cell growth, angiogenesis, cell adhesion, cell motility, and invasion [5]. NFκB is a nuclear transcription factor, a family of five proteins (RelA, RelB, c-Rel, p100, and p150), that participates in cellular functions such as proliferation, survival, and cell death and is also associated with inflammation and cancer immunoregulation [6]. COX-2 is another protein that substantially promotes cancer growth by inhibiting apoptosis, immune surveillance, inducing angiogenesis, and metastasis [7]. Among the diagnostic and prognostic biomarkers, miRNAs are of prime importance. miRNAs are non-coding, single-stranded RNA molecules that regulate gene expression at the post-transcriptional level. Different miRNAs and their role in the progression of OSCC, either as tumor suppressors or oncogenic miRNAs, has been very well-reviewed [8,9]. In OSCC, miRNA expression-based studies have recently increased, and many review studies have overtly listed the up- and down-regulated miRNAs. For example, miR- 34b, miR-100, miR-125b, miR- 133a, miR-218, miR-137, miR-222-3p, and miR-203 are downregulated in OSCC [9]. Examples of upregulated miRNAs in OSCC include miR-21, miR-155, miR-191, let-7i, miR-142-3p, miR-423, miR-221, miR-106b, miR-20a, and miR-16, miR-582-5p, and miR-196b [10].

Studies have been conducted to link the role of EGFR, COX-2, and miRNAs in chewing associated with OSCC in Asia specifically. Chewing components has shown to be associated with EGFR resulting in COX-2 expression in OSCC [11–14]. Given the importance of miRNA in OSCC, and EGFR signaling pathway as major cancer therapy target, there is insufficient information regarding their mechanistic association from this region. We have previously reported that most OSCC patients were male and had chewing habits for years, majorly gutka, betel quid, and areca nut [15]. Further, the prevalence of high-risk HPV (HR-HPV) was found to be very low, i.e., 3.8%, with no association with chewing habits [16]. In continuation of the same study, the goal is to address the questions linking the mechanistic expression of EGFR, NFκB, and COX-2 modulated by miRNAs, specially for these genes in OSCC in Pakistani patients, habitual of chewing with or without the involvement of HR-HPV.

## Methodology

### Ethical approval

The study was approved by the Ethical Review Board at Aga Khan University Hospital (AKUH) via its letter 4091-Pat-ERC, dated June 15, 2016, and by the QIMR Berghofer Human Research Ethics Committee (P1364). Patients who gave consent before surgery were recruited for their sample to be used for research purposes. Verbal approval was also taken before the questionnaire, and in the case of the deceased, consent and information were obtained from an immediate family member (approved by ERB, documented on questionnaire in the presence of a Pathology resident). The work was done as per the guidelines of the Declaration of Helsinki.

### Sample collection

From a total sample size of 186 in a cross-sectional study setting (January 2017-December 2017) (previously published) [15], 50 samples were selected randomly (computer-generated numbers). In the final sample size of 50, 23 were chewers, 20 were non-chewers, and seven were HR-HPV+ samples. At the time of sample selection for this phase, the type of chewing substance was kept blind until the statistical analysis. Information regarding chewing habits (BQ, AN and gutka) was obtained on the predefined questionnaire and has already been reported. The previous report from the authors found no association between chewing tobacco or mawa and OSCC, and therefore were not included in this study [15].

Inclusion criteria: People aged>18 years diagnosed and treated at AKUH in 2017.

Exclusion criteria: Samples not meeting the inclusion criteria of informed consent, scanty, poorly fixed/autolyzed tissues.

### Real-time PCR

RNA was extracted from FFPE blocks, and reverse transcription was performed (Detailed protocol in S1 File). EGFR, NFκB, and COX-2 gene expression was determined using Taqman qPCR Syber green Mastermix on ABI. Quant Studio ® 5 Real-Time machine. Primers of EGFR, NFκB, and COX-2 (Table 1, in S1 File) were bought from Eurofins. Total volume for a single reaction was 15μl, 7.5μl (final concentration 1X) of Mastermix, 0.6μl of 10μM primers (final concentration 120-150nM), 2μl of cDNA (700ng of RNA), and 4μl of nuclease-free water were added. Gene-specific amplification was confirmed with the dissociation curve for every target. Each sample was run in duplicates, and delta-delta Ct (ΔΔCt) was calculated according to the reported method [17]. Geometric mean fold change (GMFC) with 95% CI was calculated by GraphPad Prism v9.0 for the variation [18]. EGFR, NFκB, and COX-2 are not expressed in normal oral tissue, so we used a case-case study design with the reference groups to calculate fold change (FC) (Chewers vs Non-chewers). For descriptive purposes, the negative inverse of FC values (if any) was taken to show the relative gene expression [19].

### miRNA profiling and validation

To perform miRNA microarray profiling, nine samples were sent to the Ramaciotti Centre for Genomics, University of New South Wales, Sydney, Australia. These samples were divided into chewers, non-chewers, and HR-HPV + . The CEL files obtained from microarray profiling were analyzed using Affymetrix software, Transcriptome Analysis Console (TAC) 4.0.1. Five miRNAs, miR-3607-3p, miR-150-5p, miR-320a-3p, miR-222-3p, and miR-1260a, were chosen for validation with endogenous control RNU48 (S1 File) based on the highly significant p-values in profiling data, association with EGFR, NFkB, and COX-2 genes in NetAffyx database, and the dysregulation with HPV status.

### Fluorescent in situ hybridization (FISH) of EGFR

For the analysis of EGFR amplification status at DNA level, FISH was performed (on qPCR EGFR positive samples n = 13) using Spectrum Orange labeled FISH probe (Abbot Vysis EGFR probe kit – 01N35-020) (S1 File).

### COX-2 immunohistochemistry

COX-2 protein was analyzed by performing IHC (Ab-CX294-DAKO). Normal oral mucosal tissue was used as a negative control, and Inflammatory bowel disease (IBD-large bowel) tissue was used as a positive control. Samples were analyzed for cytoplasmic staining of COX-2 protein, and tissue with field >10% of staining was considered positive [20,21]. As COX-2 is not present in normal oral tissues, results were only recorded as positive and negative (<10% field). Olympus Light microscope was used to observe the slides at a magnification of 20X-400X.

### Statistical analysis

Data were analyzed using the SPSS package 19 (IBM., Rochester, U.S.A.) for association among miRNA, EGFR, NFκB, and COX-2 expression levels and patient characteristics, such as gender, age, chewing status, and tumor stage. Criteria of p < 0.05 and 95%CI were set for all the tests. Logistic regression was performed to predict the likelihood of the variables associated with the outcome. Odds ratios and their respective 95% confidence intervals (CI) were obtained for the association of EGFR, NFκB, COX-2 qPCR, and COX-2 IHC with the different chewing substances. Multiple linear regression with interaction was used to evaluate the association between FC miRNA expressions and EGFR/COX2/NFκB expression status by chewing status. Analyses were performed on log-transformed FC miRNA expression, and GMFC with 95% CI was presented.

## Results

**Fig 1:**
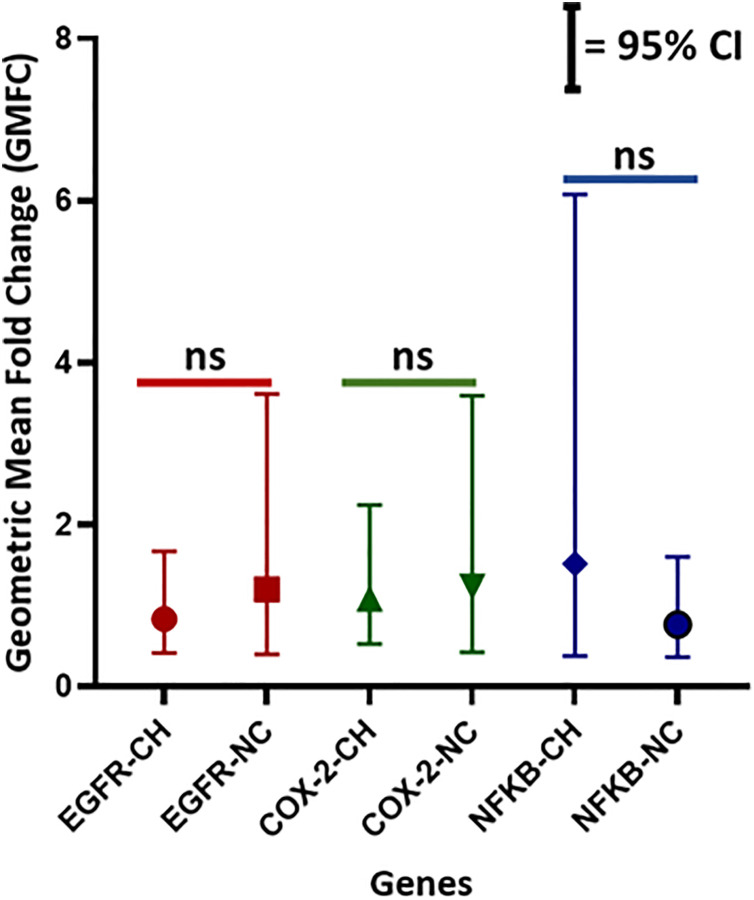
Whisker plot of GMFC with 95% CI for EGFR, COX-2, and NFκB between chewers (n = 23) and non-chewers (n = 20). Mann-Whitney U test resulted in non-significance (ns) between chewers and non-chewers for each gene expression.

mRNA expression of genes of interest in 50 samples was determined with Ct < 35. Relative quantitative expression, i.e., 2^-(ΔΔCt), was observed more in non-chewers groups, but the number of positivity was more in chewers. Results for EGFR showed the expression in 13 samples (26%) overall. When evaluated for the chewing status, 69.2% [n = 9; GMFC 95% CI, 0.83 (0.41–1.67)] were chewers, and 30.8% [n = 4; GMFC 95% CI, 1.20 (0.4–3.6)] were non-chewers. COX-2 mRNA expression was present in 17 samples overall (38%), with 64.7% [n = 11; GMFC 95% CI, 1.08 (0.52–2.24)] being chewers, 35.3% non-chewers [n = 6; GMFC 95% CI, 1.23 (0.42–3.59)]. The expression of NFκB was observed in 11 samples (22%), and 54.5% [n = 6; GMFC 95% CI, 1.51 (0.42–6.07)] were found to be chewers, whereas 45.5% were non-chewers [n = 5; GMFC 95% CI, 0.76 (0.36–1.60)]. The statistical analysis resulted in no significant difference in GMFCs between chewers and non-chewers for all genes (Fig 1).

### EGFR amplification by FISH

**Fig 2:**
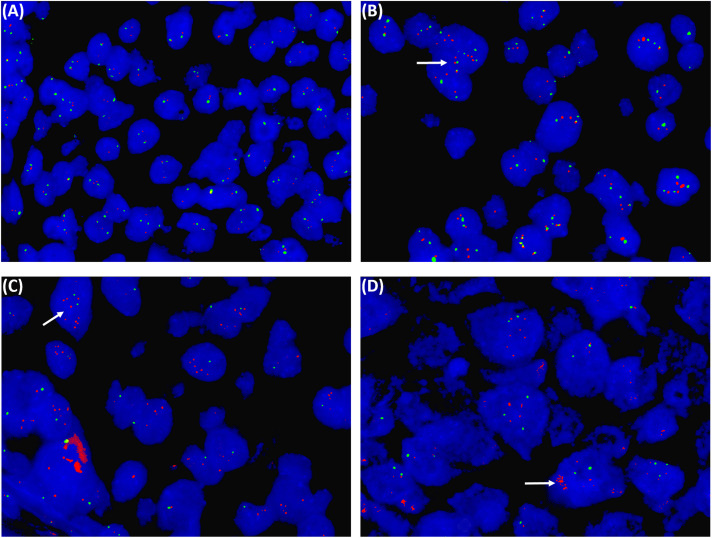
EGFR amplification by FISH, ≥ 4 EGFR signals (red) were observed, and green represents CEP7. **(A)** Negative for amplification, The arrow represents the type of amplication **(B)** EGFR gene amplification, the polysomy, **(C)** EGFR amplification with disomy, and **(D)** EGFR gene amplification as a cluster.

Only samples that were EGFR positive by qPCR (n = 13) were analyzed by FISH, and ten samples (77%) showed gene amplification. Two samples showed polysomy and increased EGFR copy number as well. In others, a mixed population was observed in terms of polysomy and disomy. Some showed disomy with ≥ EGFR signals (Fig 2A-D). Among the three non-amplified, we could not read any signal in one sample, whereas two were negative for amplification.

### COX-2 protein expression by IHC

**Fig 3:**
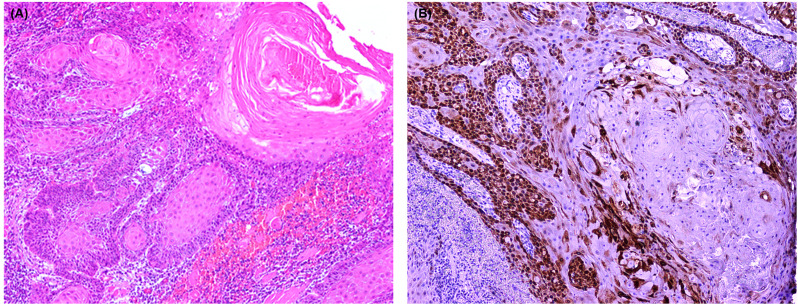
(A) H&E staining and (B) COX-2 cytoplasmic staining at 10X magnification.

**Fig 4:**
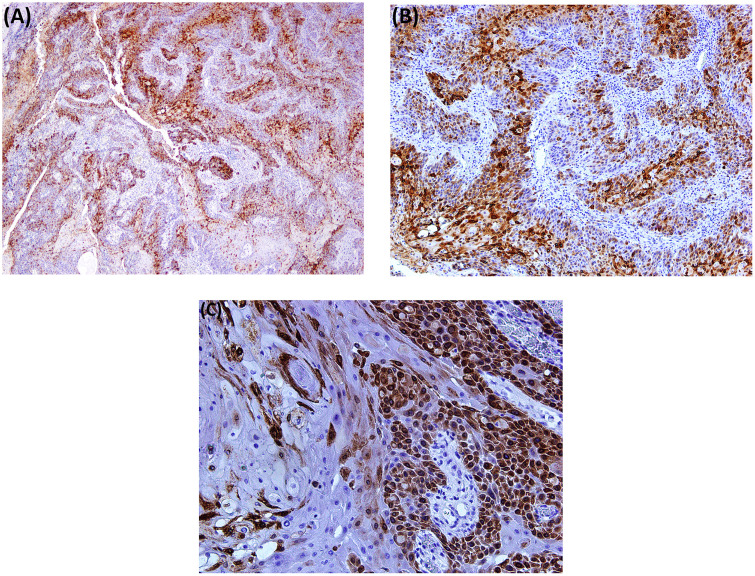
Positive COX-2 IHC cytoplasmic staining of the same sample at different magnifications, (A) 2X, (B) 10X, and (C) 20X.

With the criteria of >10% considered positive for cytoplasmic staining of COX-2 protein expression, 62% (n = 31) of the samples were positive. Strong cytoplasmic staining intensity was observed in all positive tissues (also identifiable on lower magnification, i.e., 4X). Among the positive (n = 31), 67.7% (n = 21) had a history of chewing in any form. Fig 3 (A&B) presents the IHC and their respective H&E staining cancerous tissue at 10X, and Fig 4 (A-C) represents cytoplasmic COX-2 positivity observed at increasing magnification.

### Association between EGFR., NFκB, COX-2, and chewing substances

Descriptive analysis of EGFR, NFκB, COX-2 (qPCR), and COX-2 IHC was done against chewing status (overall) and separately for BQ, gutka, and AN (Table 1 in S2 File). Most samples positive for all the molecular analyses used different chewing substances. When assessed for overall chewing status as the dependent variable, protein expression of COX-2 (IHC) was significantly associated [p < 0.05, OR: 4.50, 95% CI (1.34–15.50)]. Gene expression of EGFR and COX-2 were not significantly associated with chewing habits but presented with odds of 2.38 (95% CI: 0.6–9.0) and 1.95 (95% CI: 0.6–6.5). The model was adjusted for age, gender, EGFR, COX-2, and NFκB expression in multivariate regression analysis and with insignificant goodness-of-fit. Protein expression of COX-2 was again significantly associated with chewing habits and a high odds ratio of 4.60 (95% CI: 1.21–17.53) (Table 2 in S2 File). The association of genes and protein expression with BQ, AN, and gutka were checked individually to understand the prediction model better. When tested with BQ chewing, no variable was found to have any association, but OR>1 was observed (Table 3 in S2 File).

Univariate regression analysis of variables and AN chewing was performed, and no association was observed. Only COX-2 protein expression showed 2.7 more odds to have AN chewing [(95%CI: 0.27–25.83) (Table 4 in S2 File). Association of gutka chewing with genes and protein expression resulted in the significant association of EGFR, COX-2 mRNA, and COX-2 IHC. EGFR positivity had four times more odds of being gutka chewer (p = 0.03; 95% CI: 1.10–16.18). COX-2 gene expression was also five times more likely to be gutka chewer (p = 0.01; 95% CI: 1.38–18.57). Protein expression of COX-2 presented six times higher odds of gutka chewing. All the significant variables in univariate analysis, i.e., EGFR, COX-2 gene, and COX-2 protein, turned out to be insignificant, retaining their comparatively high ORs (Table 5 in S2 File).

### miRNA profiling and validation

**Fig 5:**
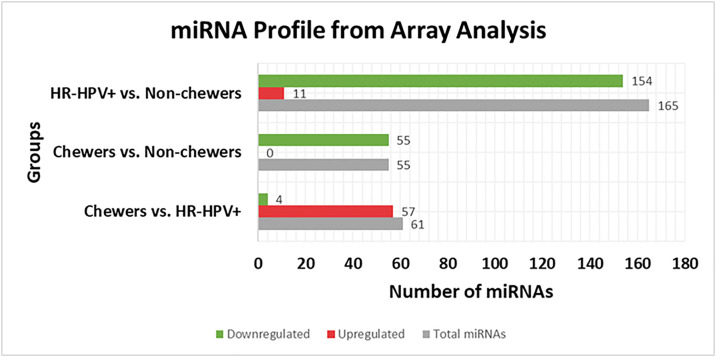
Graphic illustration of differentially expressed miRNAs in OSCC comparison groups observed on the array (FC < −2 or >2.0, p < 0.05). **Upregulation and Downregulation are presented based on the FC of the first set in each comparison group (Figure generated by TAC software).** FC = Fold change.

miRNA profiling of nine samples in groups of three resulted in differential expression of 281 mature hsa-miRNAs after the set criteria (FC < −2 or >2, and p < 0.05). In the comparison group, Chewers vs HR-HPV + ve, 61 miRNAs were observed, 55 were in Chewers vs Non-chewers, and 165 were in the group HR-HPV + ve vs Non-chewers (Fig 5). All the miRNAs obtained were analyzed by NetAffyx™ for their validated target of COX-2, EGFR, and NFκB. miRNAs targeting COX-2 were miR-146a-5p, miR-143-3p, miR-320a-3p, let-7a-5p, miR-16-5p, and let-7c-5p. EGFR-associated miRNAs included miR-145-5p, miR-155-5p, miR-16-5p, and miR-30a-5p. Validated miRNAs against p65 (REL-A) included miR-320a-3p and miR-16-5p against NFκB1. miRNA 1260a, a less reported miRNA, was significantly differentially expressed in all the groups, hence weas included for validation.

Validation of five miRNAs, miR-3607-3p, miR-150-5p, miR-320a-3p, miR-222-3p, and miR-1260a was done with qPCR. Fig 2 (S2 File) compares the relative expression of these miRNAs across three groups, normalized to the normal tissue sample. For the normal tissue, after the calculation of ΔΔCt, the FC was equal to one [16]. Chewers showed the highest FC of expression for four miRNAs, followed by non-chewers. The HR-HPV+ group showed uppermost FC (5.3) only for miR-222-3p. miR-3607-3p was downregulated in all three groups as compared to normal tissue.

### Association of miRNAs and genes with the additional status of chewing

The link between miRNAs and EGFR, COX-2, and NFkB between chewers and non-chewers was examined. miR-222-3p showed a significant association with EGFR positivity in chewers (p = 0.011). FC expression was significantly lower in EGFR +ve (GMFC 0.93, 95% CI: 0.40–2.13) compared to EGFR-ve (GMFC 5.52, 95% CI: 2.80–10.90) among chewers (p = 0.002), while there was no substantial FC expression difference between EGFR–ve and +ve among non-chewers (Table 1). Expression of miRNA 3607-3p was below the detection limits in this group, hence not reported.

**Table 1:** Geometric mean fold change (GMFC) miRNA expression by EGFR expression with the added status of chewing habits.

		Non-Chewers			Chewers			Interaction p-value***
		EGFR -ve	EGFR +ve		EGFR -ve	EGFR +ve		
miRNAs	N	GMFC (95% CI)	GMFC (95% CI)	p*	GMFC (95% CI)	GMFC (95% CI)	p**	
150-5p	40	2.59(1.50-4.47)	4.09(1.16-14.42)	0.51	4.64(2.47-8.71)	3.24(1.57-6.71)	0.46	0.33
320a-3p	32	4.75(2.61-8.63)	3.31(0.72-15.20)	0.66	2.91(1.47-5.75)	7.25(3.21-16.37)	0.089	0.19
222-3p	30	2.75(1.52-4.95)	4.40(1.36-14.28)	0.47	5.52(2.80-10.90)	0.93(0.40-2.13)	0.002	0.011***
1260a	41	3.79(2.28-6.31)	3.07(0.99-9.57)	0.73	5.24 (3.04-9.05)	4.26(2.29-7.94)	0.62	0.995

-*p and p** are based on the comparison between EGFR-ve and EGFR +ve in non-chewer and chewer, respectively.

-***interaction p < 0.05 (multiple linear regression) indicates that the association between miRNA expression and EGFR status is different between non-chewer and chewer

Association between miRNAs and COX-2 gene expression was determined with the chewing habits (Table 2). miR-222-3p was again significantly associated with COX-2 status in chewers (p = 0.007) with considerable downregulation in COX-2 positive cases (GMFC 1.09, 95% CI: 0.50–2.36, p = 0.003). miR-320a-3p was also found to be marginally associated with COX-2 expression (p = 0.054). miR-320a-3p expression was ~ 3times upregulated in COX-2 + ve cases among the chewers (GMFC COX-2 –ve = 2.73 and COX-2 + ve = 6.93) whereas, ~ 2 times downregulation was observed in COX-2 + ve in non-chewers (GMFC COX-2 –ve 5.38 and COX-2 + ve 2.82). Other miRNAs did not show any notable significance in FC with COX-2 gene expression.

**Table 2:** miRNA expression-geometric mean fold change (GMFC) by COX-2 expression with added status of chewing habits.

		Non-Chewers			Chewers			Interaction p-value***
		COX2 -ve	COX2 + ve		COX2 -ve	COX2 + ve		
miRNAs	N	GMFC (95% CI)	GMFC (95% CI)	p*	GMFC (95% CI)	GMFC (95% CI)	p**	
3607-3p	21	0.58(0.23-1.47)	0.32(0.04-2.59)	0.59	0.52(0.12-2.29)	0.60(0.16-2.28)	0.87	0.61
150-5p	40	2.65(1.44-4.86)	3.11(1.27-7.61)	0.76	4.80(2.48-9.28)	3.24(1.62-6.47)	0.41	0.44
320a-3p	32	5.38(2.84-10.16)	2.82(0.98-8.10)	0.29	2.73(1.35-5.52)	6.93(3.29-14.63)	0.073	0.054
222-3p	30	2.56(1.34-4.90)	4.19(1.68-10.48)	0.38	6.01(2.91-12.40)	1.09(0.50-2.36)	0.003	0.007***
1260a	41	4.44(2.54-7.77)	5.09(2.91-8.90)	0.23	5.09(2.91-8.90)	4.49(2.50-8.05)	0.76	0.47

-*p and p** are based on the comparison between COX-2-ve and COX-2 + ve in non-chewer and chewer, respectively.

-***interaction p-value < 0.05 (Multiple linear regression) indicates there is evidence that the association between miRNA expression and COX-2 status is different between non-chewer and chewer.

Lastly, a similar analysis was run to study the association between miRNAs and NFκB with chewing habits (Table 3). miR-222-3p was again significantly associated with NFκB between chewers and non-chewers (interaction p = 0.02) with low expression in NFκB + ve cases among the chewers (GMFC 0.87, 95% CI: 0.34–2.24, p = 0.006). miR-320a-3p did not show any significant association with NFκB status, but a considerably high expression was observed among NFκB + ve in chewers. Despite insignificance, miR-150-5p showed a relatively high expression among NFκB + ve cases in both chewers (GMFC NFκB-ve 3.93 and NFκB + ve 4.14) and non-chewers (GMFC NFκB-ve 2.18 and NFκB + ve 5.50). None of the miRNAs were significantly different with HR-HPV + ve and –ve groups. However, miR-3607-3p was downregulated in both groups and comparatively high expression all other four miRNAs was observed in HR-HPV-ve samples. (Table 6 in S2 File).

**Table 3:** miRNA expression (geometric mean fold change) by NFκB expression relating to the additional status of chewing habits.

		Non-Chewers			Chewers			Interaction p-value***
		NFκB -ve	NFκB + ve		NFκB -ve	NFκB + ve		
miRNAs	N	GMFC (95% CI)	GMFC (95% CI)	p*	GMFC (95% CI)	GMFC (95% CI)	p**	
3607-3p	21	0.59(0.23-1.54)	0.36(0.07-1.84)	0.58	0.35(0.10-1.25)	1.03(0.25-4.30)	0.25	0.23
150-5p	40	2.18(1.24-3.86)	5.50(2.12-14.25)	0.10	3.93(2.31-6.69)	4.14(1.60-10.74)	0.92	0.27
320a-3p	32	4.17(2.23-7.78)	7.74(1.58-37.97)	0.46	3.93(2.05-7.52)	5.07(1.85-13.86)	0.67	0.72
222-3p	30	2.80(1.48-5.31)	3.69(1.28-10.66)	0.65	4.78(2.44-9.34)	0.87(0.34-2.24)	0.006	0.024
1260a	41	3.54(2.09-6.00)	4.13(1.54-11.08)	0.78	4.89(3.03-7.89)	4.54(2.03-10.16)	0.87	0.75

-*p and p** are based on the comparison between NFκB –ve and NFκB + ve in non-chewer and chewer, respectively

--***interaction p-value < 0.05 indicates (Multiple linear regression) there is evidence that the association between miRNA expression and NFκB status is different between non-chewer and chewer

## Discussion

EGFR, NFκB, and COX-2 belong to the gene group with multiple roles in the initiation and progression of OSCC. In many cancers, expression or overexpression of EGFR is among the etiological factors. In our study, the overall EGFR expression was seen in around a quarter of samples, with the majority being chewers. EGFR gene amplification is the most common phenomenon reported in the OSCC, and low mutation rate (0.58%) in BQ associated OSCC [22]. Studies from this region have reported increased expression of EGFR in OSCC [23,24]. It has also been shown to be an independent OSCC driver or associated with downstream inflammatory biomarkers [25].

Some of the downstream inflammatory targets of EGFR are NFκB and COX-2, and their overexpression is an essential factor in many cancers, including OSCC, irrespective of the primary cause [26]. This study determined the transcriptionally active COX-2 gene (qPCR) and the protein expression (IHC) to be 34% and 62%, respectively, with the majority being chewers. Different studies on OSCC have observed varied protein (IHC) expression of 77.8% [27], 41.7% [28], 34% [29], and 70% [30]. It is well-known that rampant chewing habits, tobacco use (smoking/SLT), and alcohol consumption are the foremost causes of OSCC. When evaluated for separate chewing substances (BQ, AN, and gutka), EGFR, COX-2 gene, and protein expression were significantly associated with gutka chewing. Other studies suggest that reactive oxygen species (ROS) generated during the metabolism of these products could activate EGFR, which in turn could activate epithelial aging, Src/Ras, MAPKs, Wnt, or NFκB activation via the PI3K/Akt pathway [11,31–33]. EGFR and NFκB independent COX-2 expression among chewers proposes a direct effect of SLT compounds on the COX-2 gene, which correlates with some earlier reports [24,31,34,35]. The insignificant association in multivariate model is indicative of their independent importance in the OSCC. Secondly, due to less sample size, the expression of a single gene could be masked by the others, and limited study power could result in false negatives.

Next, miRNAs were evaluated as they have been associated with carcinogenesis. Some of the miRNAs have previously been validated in HNSCC. or OSCC., e.g., miR-21, miR-155, miR-199a, let-7, miR-99a, miR-125, miR-15b, miR-185 [36,37]. In the comparison groups of this study, HPV + ve showed downregulation for the bulk of miRNAs when compared with chewers and non-chewers; this was further confirmed in the real-time validation. The likely reason for this down-regulation in HPV + ve samples could be epigenetic changes, like hyper-methylation [38,39]. In the comparison group of chewers vs. non-chewers, the majority were downregulated in chewers. There could be many reasons for this, but the genotoxicity of the chewing compounds could be one explanation. As reviewed, DNA damage, oxidative stress, miRNA adducts, and miRNA sensitivity resulting from chemical insult can cause the dysregulation of miRNA expression [40,41].

In the validation of five miRNAs (hsa-miR-222-3p, hsa-miR- 320a-3p, hsa-miR-1260a, hsa-miR-3607-3p, and hsa-miR-150-5p), the highest expression was observed in chewers for all the miRNAs but hsa-miR-222-3p. Its downregulation has been reported in HPV + ve HNSCC cell lines [42,43]. In another study, small RNA sequence analysis of miRNAs in OSCC provided significant differential expression of hsa-miR-222-3p between chewers and non-chewers. However, HR-HPV + ve samples were not included in this study [44]. This leaves the role of this miRNA to be explored extensively in HPV-related and unrelated OSCC. On contrary, its upregulation has also been reviewed in oral cancer pathogenesis [45].

miR-150-5p was upregulated in the samples compared to normal tissue. Conflicting reports are present for the dysregulation of miR-150-5p. It has been shown to be upregulated in OSCC, HNSCC [45,46], and gastric cancers [47], whereas downregulation is reported in other types of cancers [48]. Depending on the tissue type, this could be credited to miRNA multifunctionality as an oncogene and tumor suppressor. miRNA- 3607-3p was significantly downregulated in all three groups compared to normal tissue. Low expression of this miRNA has been reported for prostate cancer [49], NSCLC [50] and pancreatic cancer [51]. These studies also reported increased cancer progression with low expression, implying miR-3607-3p as a tumor suppressor. As molecular mechanisms in cancer biology could be similar in different cancers, these mechanisms could be hypothesized for OSCC.

Dysregulation of miR-320a in multiple malignancies has previously been reported. Its decreased expression associated with angiogenesis has been found for OSCC [52]. However, an upregulation (50-fold) of another member of this miRNA’s family (miR-320b) is reported in serum samples of OSCC patients [53] that seconds the upregulated of miR-320a-3p in this study. The differential expression of a somewhat novel miR-1260a was observed in our study. This study presents its upregulation in OSCC as compared to normal tissue. This immune system-related miRNA is extensively expressed by activated B-cells [54]. Its upregulation has been reported in malignant melanoma [55], bladder cancer [56], breast cancer, ovarian cancer [57], and prostate cancer [58]. Its role in oral carcinogenesis has not yet been explored, so our results could be the first to provide future insight into this miRNA’s potential role in oral carcinogenesis. During the validation assay, some samples did not give Ct < 35 for specific miRNAs, which resulted in a somewhat reduced number. This could be due to miRNA degradation during harsh FFPE processing, leaving expression beyond the limit of detection for that particular miRNA.

Further, the expression of the miRNAs with respect to EGFR, NFκB, and COX-2 and chewing status was evaluated. The exact mechanism of the inconsistent expression of miRNAs is unknown about chewing, mainly because of the number of substances in SLT/areca nut, hundreds of other miRNAs, and tissue types. Some studies have reported the effect of tobacco/cigarette compounds on the miRNA profile of oral cancer. A decrease in the expression of miR-1266-5p and miR-196b-5p and an increase in the expression of epithelial-mesenchymal transition related proteins have been observed in tobacco-treated oral keratinocytes [59]. miR-193a-5p is downregulated in smokers due to DNA hypermethylation [60]. Tobacco carcinogens have been reported to induce epigenetic silencing of miRNAs by trimethylation of H3K27 (Histone 3, lysine 27^th^ amino acid) in lung cancer [61]. ROS could be another factor that regulates miRNA expression in chewers. ROS can achieve this either by decreasing DICER expression, increasing expression of transcription factors like NFκB, p53, and epigenetic changes [62]

Another study on colon cancer reported EGFR-regulated suppression of miR-143 and miR-145 [63]. Sometimes, through a complex negative feedback loop, genes being targeted by miRNA inhibit the miRNA itself [64]. In this study, miR-222-3p was not downregulated in tumor tissue compared to normal tissue but between the evaluation groups of chewers and non-chewers. It is speculated that miR-222-3p might follow one of the said mechanisms and play an essential role in molecular pathogenesis, considering chewing SLT/AN. The observed downregulation of miR-222-3p in EGFR/NFκB/COX-2 positive chewers and upregulation in EGFR/NFκB/COX-2 positive non-chewers, suggests a role both as a tumor suppressor and oncomir, respectively. Likewise, miR-320 expression could be affected by tobacco-associated compounds like Benzo(a)pyrene (BaP). According to the UCSC Genome Browser, BaP interacts with miR-320 (from Comparative Toxicogenomics Database (CTD)). A study conducted on murine bronchial epithelial cells showed that a 1μM dose of BaP for 24h enhanced the miR-320 expression, which caused G1 cell cycle arrest [65]. miR-320a can also regulate COX-2 through ERK/NFκB pathway [66]. Genetic alterations (SNP) in the 3’-UTR of COX-2 have also been shown to affect miRNA activity, leading to COX-2 overexpression [67]. It is anticipated that chewing substances could alter the miR-320 expression overtly with COX-2 to promote tumorigenesis. Similarly, the role of miR-1260a needs to be explored in oral cancer.

## Conclusion and future implications

In conclusion, this study showed that EGFR, NFkB, COX-2, and miRNAs expression could be associated with OSCC with chewing habits irrespective of HR-HPV involvement. Transcriptional or translational expression of COX-2 independent of EGFR and/or NFκB signifies its unequivocal role in chewing-mediated OSCC miRNAs were found to have differential expression in chewers, non-chewers, and HR-HPV in microarray analysis. This is the first study to explore the collaborative role of HR-HPV, EGFR, NFκB, COX-2, and miRNAs in OSCC with rampant chewing habits in Pakistan. A limitation of this study is the relatively small sample size, leading to a lack of statistical analysis and conclusions robustness. Another limitation is that we could only do miRNA profiling on nine samples which limits the conclusions and generalisability of the miRNA analysis. This study could be extended to dissect the role of miRNA related to the EGFR signaling pathway in larger sample size and in vivo and in vitro, specifically for OSCC. miRNA expression and Tyrosine Kinase pathway could potentially be used in a tool for prediction of prognosis for oral cancer patients in the future. Since our study is exploratory, further research is needed.

## Supporting information

S1 File Methods. (DOCX)

S2 File Results. (DOCX)
