# Supplementary material for: Analysis of EGFR signaling pathway; miRNAs and inflammatory biomarkers in a high-risk oral cancer population in Pakistan - An exploratory study
Source: PLoS One. 2026 Feb 23;21(2):e0340264. doi: 10.1371/journal.pone.0340264 (PMC12928398; doi:10.1371/journal.pone.0340264)
Supplement: S1 File — (DOCX) [file pone.0340264.s001.docx]

**Supplementary File 1**

**Methodology**

**RNA Extraction**

RNeasy FFPE Kit from Qiagen (Cat#73504) was used to extract the whole RNA as per the manufacturer's protocol with minor modifications. Three sections of 6-8μm were collected in a clean 1.5 ml Eppendorf tube and deparaffinized for RNA extraction. Pellets were air-dried, and 150μl PKD buffer and 10μl Proteinase K were added. Tubes were briefly spun and were incubated at 56^o^C for 45 mins to obtain maximum protein lysis. The rest of the process was as per the manufacturer's protocol. For RNA elution, 30μl of RNase-free water was added directly to the spin column and incubated at room temperature for 15-20mins. Tubes were centrifuged at full speed for 1 min, and the same flow-through was applied again to the column to have a better RNA yield. RNA quantification was performed by measuring 260/280 OD with Nanodrop Spectrometer (A260/280>1.9 was used to indicate RNA purity).

**cDNA synthesis**

Reverse transcription of RNA was performed using iScript ™ Reverse Transcription Supermix (Cat#170-881). In a total volume of 20μl per reaction, 4μl of RT-Supermix and 700ng of RNA were added, and thermal cycler conditions followed were: Priming at 25^o^C for 5 mins, Reverse transcription at 46^o^C for 20 mins, and RT-inactivation at 95^o^C for 1 min.

Sequences were obtained against mRNA transcripts, β-Actin-NM_001101.5 [1] , EGFR-NM 005228.3 [2], NFκB-NM_001145138.2, and COX-2 -NM_000963.4 [3]

**Table 1: Primer sequences of EGFR, NFκB, COX-2, and Actin for qPCR**

|  | **Gene** | **Seq 5’->3’** | **Location on Chr** | **Priming Temp ^o^C** |
| --- | --- | --- | --- | --- |
| 1. | EGFR-F | GGCAGGAGTCATGGGAGAA | 2034-2052 | 58 |
|  | EGFR-R | GCGATGGACGGGATCTTAG | 2168-2186 |  |
| 2. | NFκB-F | ATCCCCATCCTCCAGCTTCT | 2028-2047 | 60.5 |
|  | NFκB-R | GCCATACAGGGGCTGGTATC | 2160-2179 |  |
| 3. | COX-2-F | CCCTTGGGTGTCAAAGGTAA | 575-594 | 58.5 |
|  | COX-2-R | GCCCTCGCTTATGATCTGTC | 724-743 |  |
| 4. | Actin-F | AGAGCTACGAGCTGCCTGAC | 797-816 | 60 |
|  | Actin-R | AGCACTGTGTTGGCGTACAG | 980-961 |  |

**miRNA Microarray and analysis**

With 30,424 probe sets, Affymetrix miRNA array 4.0 provides profiling of ~2500 mature Human miRNAs across one sample. Comparison analysis was run in 3 settings, i.e., Chewers vs Non-chewers, Chewers vs HR-HPV+, and Non-chewers vs HR-HPV+. The expression analysis settings were RMA+DABG (Human Only), using the ebayes ANOVA method, miRNA FC<-2 or >2, and p< 0.05. miRNAs were analyzed for their sequences, properties, and target genes in the miRNA databases covered by NetAffyx^TM^.

**Real-Time PCR validation of miRNAs:**

Based on highly significant p-values or if associated with EGFR/COX-2 in the NetAffyx database, five miRNAs, miR-3607-3p, miR-150-5p, miR-320a-3p, miR-222-3p, and miR-1260a, were chosen for validation with endogenous control RNU48. cDNA synthesis (Taqmant miRNA reverse transcription kit -4366596) was performed (Supplementary File 1) in 15μl per reaction, and the master mix was prepared by adding 6.75μl of RT Primer pool, 0.34 μl of 100mM dNTPs, 3.38μl MultiScribe Reverse Transcriptase, 1.69μl 10X RT buffer, 0.21μl Rnase Inhibitor, and 1.14μl of Nuclease-free water. In each PCR tube, 12μl master mix and 3μl of RNA (concentration of 700ng) were added, and tubes were incubated on ice for 5 mins. In Veriti™ Thermal Cycler (ABI), the thermal program was run at 16^o^C for 30mins, 42^o^C for 30 mins, and 85^o^C for 5 mins.

The qPCR reaction mix was prepared by adding 0.1μl RT product, 5μl TaqMan Universal Master Mix II (2X), and 4.42μl of Nuclease free water per reaction (total 9.5μl.) Then, 0.5μl of each 20X TaqMan miRNA assay was added separately into each tube. In Quant Studio® 5 Real-Time machine, tubes were run with thermal cycler conditions; 95^o^C for 10 mins followed by 40 cycles at 95^o^C for 15 sec and 60^o^C for 1 min.

***Analysis of miRNA***

miRNA expression in normal tissue was considered a control and used as a reference for calculating ΔΔCt and FC. Samples with Ct<35 were considered positive, and GMFC with 95%CI was calculated for statistical analysis. Relative expression was articulated as the difference in expression of a miRNA in a particular group compared to the control.

**Fluorescent In Situ Hybridization (FISH) of EGFR**

Briefly, 4-5µm thick sections were taken on charged glass slides. After paraffin removal by xylene, sections were pretreated in 2mM NaCN for 30-55 mins and later held in pepsin for an average of 30-40 min, followed by denaturation in formamide solution. To each section, 10µl of DNA probe cocktail containing CEP7 (green signal) and LSI EGFR (red signal) was applied, coverslipped, and sealed for overnight hybridization at 37°C in a humidified chamber. Afterwards, slides were washed and counter-stained with DAPI. Slides were observed for EGFR probe signals at 100X magnification using a Nikon Eclipse-epi-fluorescent microscope (Nikon, Tokyo, Japan). Red and green signals emanating from the stained sections were counted as Colorado Scoring criteria, i.e., ≥4signals in ≥ 10% of the analyzed cell [4]. The analysis was recorded as “amplified” (low amplification and high amplification/polysomy with EGFR signal) and “non-amplified” (normal signals-2 per cell).

References:

1. Weber D, Zhang M, Zhuang P, Zhang Y, Wheat J, Currie G. The efficacy of andrographolide and its combination with betulinic acid in the treatment of triple-negative breast cancer. Cancer Therapy & Oncology International Journal. 2017;4(1):1-10.

2. Chen G, Kronenberger P, Teugels E, De Grève J. Influence of RT-qPCR primer position on EGFR interference efficacy in lung cancer cells. Biological procedures online. 2010;13:1-8.

3. Xu X, Lan W, Jin X, Wang B, Yan H, Chen X, et al. Regulated expression of PTPRJ by COX-2/PGE2 axis in endothelial cells. Plos one. 2014;9(12):e114996.

4. Varella-Garcia M, Diebold J, Eberhard D, Geenen K, Hirschmann A, Kockx M, et al. EGFR fluorescence in situ hybridisation assay: guidelines for application to non-small-cell lung cancer. Journal of clinical pathology. 2009;62(11):970-7.
