# Supplementary material for: Analysis of EGFR signaling pathway; miRNAs and inflammatory biomarkers in a high-risk oral cancer population in Pakistan - An exploratory study
Source: PLoS One. 2026 Feb 23;21(2):e0340264. doi: 10.1371/journal.pone.0340264 (PMC12928398; doi:10.1371/journal.pone.0340264)
Supplement: S2 File — (DOCX) [file pone.0340264.s002.docx]

**Supplementary File 2 (Results)**

Table 1: Descriptive analyses of positive cellular and molecular biomarkers for chewing (overall), betel quid, areca nut, and gutka

| **Total**  **N=50** | **Chewing overall**  **n(%)** | | **Pan**  **n(%)** | | **Areca Nut**  **n(%)** | | **Gutka**  **n(%)** | |
| --- | --- | --- | --- | --- | --- | --- | --- | --- |
|  | **No** | **Yes** | **No** | **Yes** | **No** | **Yes** | **No** | **Yes** |
| EGFR  Positive n=13 | 4(30.8) | 9 (69.2) | 6 (46.2) | 7(53.8) | 12(92.3) | 1(7.7) | 6(46.2) | 7(53.8) |
| COX-2  Positive n=17 | 6(35.3) | 11(64.7) | 10(58.8) | 7(41.2) | 16(94.1) | 1(5.9) | 8(47.1) | 9(52.9) |
| NFκB  Positive n=11 | 5(45.5) | 6(54.5) | 6(54.5) | 5(45.5) | 11(100) | 0 | 6(54.5) | 5(45.5) |
| COX-2 IHC  Positive n=31 | 10(32.3) | 21(67.7) | 17(54.8) | 14(45.2) | 27(87.1) | 4(12.9) | 18(58.1) | 13(41.9) |

-“n” represents the total number of positive samples for one gene or protein biomarker.

Table 2: Univariate and multivariate regression model of genes and overall chewing products

| **Variables** | **Chewing overall** | | **Univariate analysis** | | **Multivariate analysis** | |
| --- | --- | --- | --- | --- | --- | --- |
|  | **Yes-no(%)** | **No-n(%)** | **OR (95% CI)** | **p-value** | **Adjusted OR(95% CI)** | **p-value** |
| **Age (Mean±SD**) | 50.1±14.5 | 49.3±13.9 | 1.00(0.96-1.04) | 0.83 |  |  |
| **Gender** | | | | | | |
| Female | 7(50) | 7(50) | 1 (Reference) | | | |
| Male | 20(55.6) | 16(44.4) | 1.25(0.36-4.31) | 0.72 |  |  |
| **EGFR** | | | | | | |
| Negative | 18(48.6) | 19(51.4) | 1 | | | |
| Positive | 9(69.2) | 4(30.8) | 2.38(0.62-9.09) | 0.21* | 2.90(0.28-30.90) | 0.36 |
| **COX-2 (mRNA)** | | | | | | |
| No | 16(48.5) | 17(51.5) | 1 | | | |
| Yes | 11(64.7) | 6(35.3) | 1.95(0.58-6.51) | 0.28 |  |  |
| **NFκB** | | | | | | |
| No | 21(53.8) | 18(46.2) | 1 | | | |
| Yes | 6(54.5) | 5(45.5) | 1.00(0.27-3.94) | 0.97 |  |  |
| **COX-2 IHC** | | | | | | |
| No | 6(31.6) | 13(68.4) | 1 | | | |
| Yes | 21(67.7) | 10(32.3) | 4.50(1.34-15.50) | 0.015* | 4.60 | 0.02* |

-The model was adjusted for age, gender, EGFR, COX-2 mRNA, and NFκB due to the biological importance of variables, p<0.05 significant, OR 1 as reference.

-*represents the variables in the univariate model were adjusted in the multivariate regression model.

Table 3: Logistic regression model of Betel quid use as a dependent factor with gene and protein expression

| **Variables** | **Betel quid use** | | **Univariate analysis** | |
| --- | --- | --- | --- | --- |
|  | **Yes-n(%)** | **No-n(%)** | **OR (95% CI)** | **p-value** |
| **Age** (Mean±SD) | 50.22±15.41 | 49.56±13.63 | 1.00(0.96-1.05) | 0.87 |
| **Gender** | | | | |
| Female | 7(50) | 7(50) | 1(Reference) | |
| Male | 20(55.6) | 16(44.4) | 1.60(0.41-6.07) | 0.49 |
| **EGFR** | | | | |
| Negative | 18(48.6) | 19(51.4) | 1 | |
| Positive | 9(69.2) | 4(30.8) | 2.70(0.75-10.13) | 0.13* |
| **COX-2 mRNA** | | | | |
| No | 16(48.5) | 17(51.5) | 1 | |
| Yes | 11(64.7) | 6(35.3) | 1.40(42-4.68) | 0.58 |
| **NFκB** | | | | |
| No | 21(53.8) | 18(46.2) | 1 | |
| Yes | 6(54.5) | 5(45.5) | 1.60(0.43-6.49) | 0.46 |
| **COX-2 IHC** | | | | |
| No | 6(31.6) | 13(68.4) | 1 | |
| Yes | 21(67.7) | 10(32.3) | 3.00(0.83-11.44) | 0.09* |

-p<0.05 and OR 1 as reference. No significance was observed with any variable in Multivariate analysis, hence not reported in the table

-* Model was adjusted for the variables p<0.2 in univariate model

Table 4: Logistic regression analysis of Areca nut with gene and protein expressions as independent factors

| **Variables** | **Areca Nut use** | | **Univariate analysis** | |
| --- | --- | --- | --- | --- |
|  | **Yes-n (%)** | **No-n (%)** | **OR (95% CI)** | **p-value** |
| **Age** (Mean±SD) | 59±24 | 48.78±12.63 | 1.05(0.98-1.12) | 0.14* |
| **Gender** | | | | |
| Female | 3(21.4) | 11(78.6) | 1 (Reference) | |
| Male | 2(5.6) | 34(94.4) | 0.21(0.032-1.46) | 0.12* |
| **EGFR** | | | | |
| Negative | 4(10.8) | 33(89.2) | 1 | |
| Positive | 1(7.7) | 12(92.3) | 0.45(0.70-6.78) | 0.75 |
| **COX-2 mRNA** | | | | |
| No | 4(12.1) | 29(87.9) | 1 | |
| Yes | 1(5.9) | 16(94.1) | (0.04-4.41) | 0.49 |
| **NFκB** | | | | |
| No | 5(12.8) | 34(87.2) | 1 | |
| Yes | 0 | 11(100) | - | 0.99 |
| **COX-2 IHC** | | | | |
| No | 1(5.3) | 18(94.7) | 1 | |
| Yes | 4(12.9) | 27(87.1) | 2.66(0.27-25.83) | 0.39 |

-p<0.05 and OR 1 as reference. As no significance was observed with any variable in Multivariate regression analysis, so not reported in the table.

-* represents variables adjusted in multivariate model.

Table 5: Univariate and multivariate logistic regression analysis of Gutka use and genes and protein expression

| **Variables** | **Gutka use** | | **Univariate analysis** | | **Multivariate analysis** | |
| --- | --- | --- | --- | --- | --- | --- |
|  | **Yes-N(%)** | **No-N(%)** | **OR (95% CI)** | **p-value** | **Adjusted OR(95%CI)** | **p-value** |
| **Age** (Mean±SD) | 46.00±11.24 | 51.43±15.7 | 1.00(0.93-1.02) | 0.22 |  |  |
| **Gender** | | | | | |  |
| Female | 2(14.3) | 12(85.7) | 1 (Reference) | | | |
| Male | 13(36.1) | 23 63.9) | 3.40(0.65-17.56) | 0.14* | 2.20(0.36-13.93) | 0.38 |
| **EGFR** | | | | | | |
| Negative | 8(21.6) | 29(78.4) | 1 | | | |
| Positive | 7(53.8) | 6(46.2) | 4.20(1.10-16.18) | 0.03* | 1.90(0.19-19.96) | 0.57 |
| **COX-2 mRNA** | | | | | | |
| No | 6(18.2) | 27(81.8) | 1 | | | |
| Yes | 9(52.9) | 8(47.1) | 5.00(1.38-18.57) | 0.01* | 1.80(0.18-17.76) | 0.60 |
| **NFκB** | | | | | | |
| No | 10(25.6) | 29(74.4) | 1 | | | |
| Yes | 5(45.5) | 6(54.5) | 2.40(0.60-9.67) | 0.21 |  |  |
| **COX-2 IHC** | | | | | | |
| No | 2(10.5) | 17(89.5) | 1 | | | |
| Yes | 13(41.9) | 18(58.1) | 6.10(1.20-31.32) | 0.03* | 4.00(0.67-23.19) | 0.12 |

-*p-values were adjusted in the multivariate model along with age, -OR 1 is taken as reference, and p<0.05 is significant

Table 6: Geometric mean fold change (GMFC) miRNA expression by HR-HPV status

|  | **HR-HPV -ve** | | | **HR-HPV +ve** | | |
| --- | --- | --- | --- | --- | --- | --- |
| **miRNA** | **N** | **GMFC (95% CI)** | **n** | **GMFC (95% CI)** | **n** | **p-value** |
| **3607-3p** | 21 | 0.57 (0.29-1.12) | 17 | 0.43 (0.11-1.75) | 4 | 0.71 |
| **150-5p** | 40 | 3.48 (2.42-5.00) | 36 | 2.43 (0.82-7.20) | 4 | 0.53 |
| **320a-3p** | 32 | 4.71 (3.16-7.00) | 29 | 2.13 (0.62-7.33) | 3 | 0.22 |
| **222-3p** | 30 | 2.86 (1.80-4.56) | 26 | 2.84 (0.86-9.31) | 4 | 0.99 |
| **1260a** | 41 | 4.43 (3.24-6.05) | 38 | 2.59 (0.86-7.85) | 3 | 0.35 |

-p>0.05, nonsignificant

**
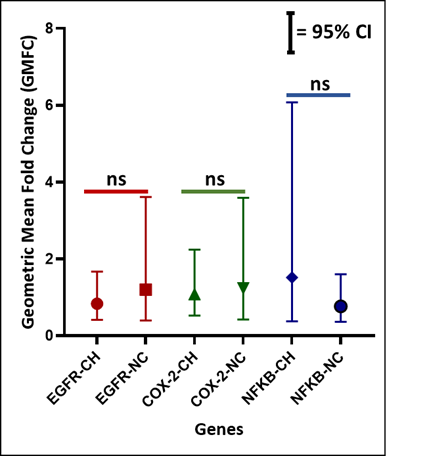
**

Figure 1: Whisker plot of GMFC with 95% CI for EGFR, COX-2, and NFB between chewers (n=23) and non-chewers (n=20). Mann-Whitney U test resulted in non-significance (ns) between chewers and non-chewers for each gene expression.

**
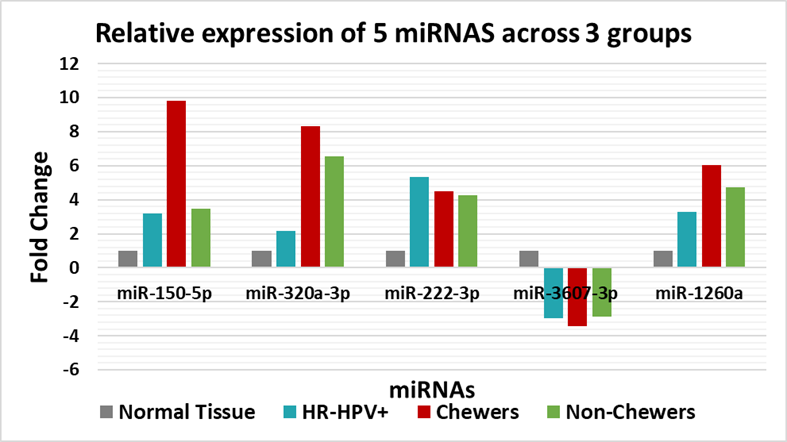
**

Figure 2: Comparison of Fold Change (FC) expression of miR-150-5p, miR-320a-3p, miR-222-3p, miR-3607-3p, and miR-1260a in normal tissue, HR-HPV+, Chewers, and Non-chewers.
